# Use of epidemiological and entomological tools in the control and elimination of malaria in Ethiopia

**DOI:** 10.1186/s12936-018-2172-1

**Published:** 2018-01-12

**Authors:** Abebe Animut, Bernt Lindtjørn

**Affiliations:** 1 Aklilu Lemma Institute of Pathobiology, Addis Ababa University, P. O. Box 1176, Addis Ababa, Ethiopia; 2 Center for International Health, University of Bergen, Bergen, Norway

**Keywords:** Malaria, Epidemiology, Entomology, Survey, Elimination

## Abstract

Malaria is the leading public health problem in Ethiopia where over 75% of the land surface is at risk with varying intensities depending on altitude and season. Although the mortality because of malaria infection has declined much during the last 15–20 years, some researchers worry that this success story may not be sustainable. Past notable achievements in the reduction of malaria disease burden could be reversed in the future. To interrupt, or even to eliminate malaria transmission in Ethiopia, there is a need to implement a wide range of interventions that include insecticide-treated bed nets, indoor residual spraying, improved control of residual malaria transmission, and improved diagnostics, enhanced surveillance, and methods to deal with the emergence of resistance both to drugs and to insecticides. Developments during the past years with increasing awareness about the role of very low levels of malaria prevalence can sustain infections, may also demand that tools not used in the routine control efforts to reduce or eliminate malaria, should now be made available in places where malaria transmission occurs.

## Overview of malaria transmission in Ethiopia

**Fig. 1:**
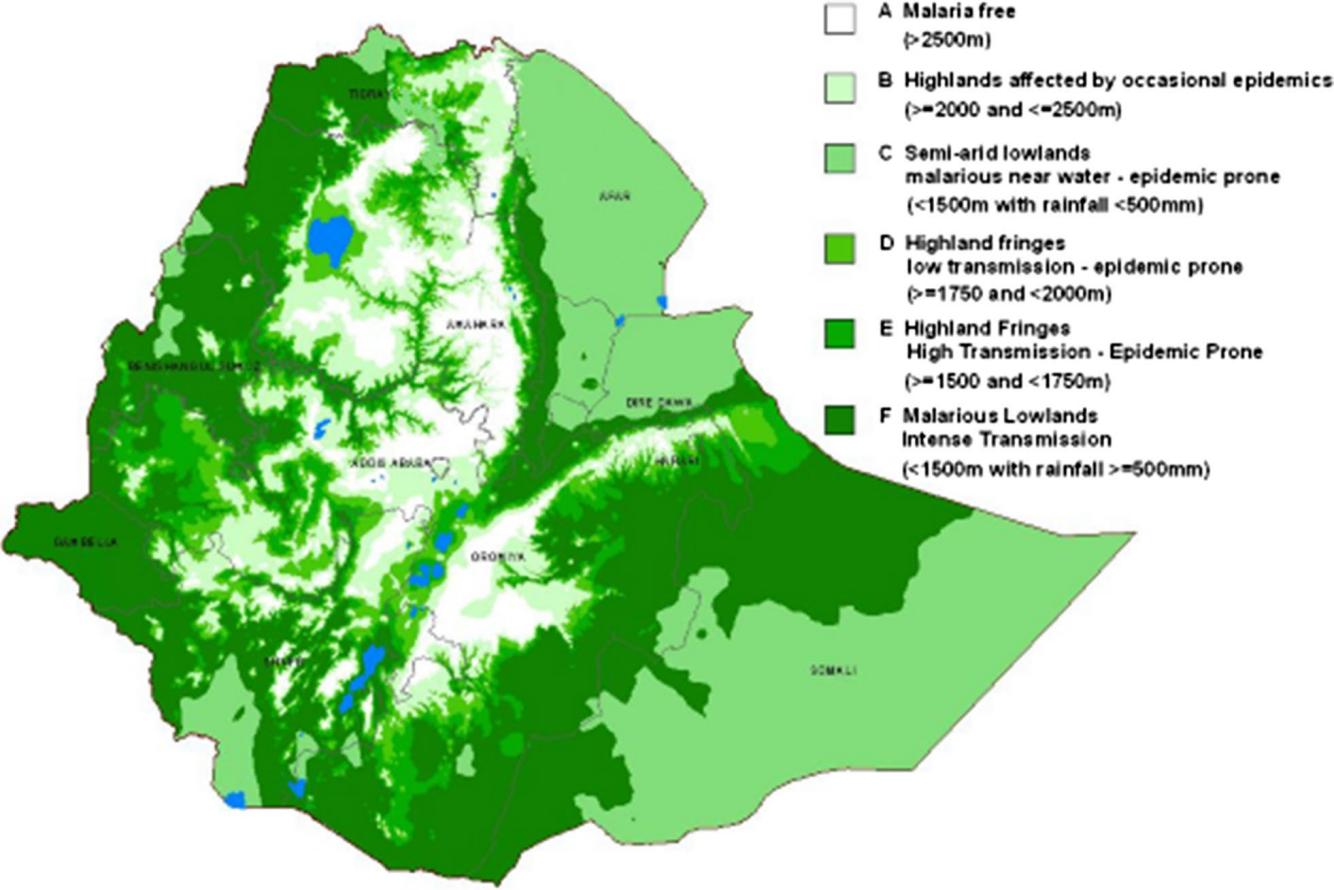
Malaria risk stratification of Ethiopia [https://www.ephi.gov.et/images/pictures/download2009/MIS-2015-Final-Report-December-_2016.pdf]

Malaria is the leading public health problem in Ethiopia where over 75% of the land surface is at risk with varying intensities depending on altitude and season (Fig. 1). Documentation of malaria transmission in Ethiopia probably began in the 1930s and was indicated to occur widely up to 2492 m above sea level in Addis Ababa, the capital city of the country [1]. In 1953, in a small area near Gondar, along Lake Tana, about 7000 deaths were estimated to occur. In the last half of the 1958, an epidemic of unusual intensity occurred throughout the highlands of the country including Showa, Gojjam, Beghemder, Wollo and portions of Wollega, Arusi, Harar and Sidamo that resulted in high mortality and morbidity (exceeding 75% of the affected population). *Plasmodium falciparum* was the predominant parasite (71%) followed by *Plasmodium vivax* (22%) and *Plasmodium malariae* 3%. Unusually higher rainfall, temperature and humidity could be the driving factors of the epidemic [2]. A longitudinal study in the period 1967–1969 in Gambella (with an estimated population of 1600) revealed, average monthly *P. falciparum* parasite rate of 58% (ranging from 29.5 to 73.5%) among children less than 15 years old and 35% (ranging from 11.4 to 40.4%) among adult 15 years of age or older. Prevalence of *P. malariae* was 16% in children and 7% in adults. In addition to these, prevalence of *Plasmodium ovale* was 2.1% and *P. vivax* was 0.9%. *Anopheles arabiensis*, *Anopheles funestus* and *Anopheles nili* were responsible for transmission of the disease [3, 4].

Large-scale epidemics were recorded at irregular intervals such as in 1981, 1988, 1991, 1992, and 1998 due to *P. falciparum* [5–7]. In 2003, malaria epidemics was observed in 25 districts of the country with an average increase of sixfold from the threshold level [8]. *Plasmodium falciparum* has been considered to be the major cause of malaria followed by *P. vivax* and the current burden of the two parasites (*P. malariae* and *P. ovale*) remains unclear. The malaria burden reduced substantially in the WHO African Region since 2010 [9] mainly due to the scaled-up use of malaria control interventions, such as long-lasting insecticide-treated mosquito nets (LLINs), indoor residual spraying (IRS), and treatment of *P. falciparum* infections with artemisinin-based combination therapy and accurate diagnosis of suspected cases [9, 10]. However, the disease occurred in an increasing trend between 2014 and 2016 in the region [9]. The rising global temperature may enhance a rise in mosquito breeding, parasite development cycle and bite frequency of vectors thereby increasing the likelihood of malaria infection [11].

Malaria causing parasites and their vectors are adaptable and capable of circumventing interventions in their nature. The deadly malaria parasite, *P. falciparum,* has developed resistance to most of the available anti-malarial drugs and its *Anopheles* vectors have developed resistance to most classes of insecticides [10, 12]. Moreover, transmission of the disease is complex and is affected by various factors including season, climate, ecology, parasite genetic polymorphisms, socio-economic status, and micro-environment [13–15]. Some people carry the parasite with no clinical manifestation while others get severe sickness and die. Although LLINs remain effective in protecting people from mosquito bites, the public health impact of vector resistance to insecticides used in the nets remains a research question [9, 16]. Thus, to maintain the declining trend of the disease and ultimately eliminate it from Ethiopia, there is a need for strengthened use of existing intervention tools and development and/or integration of a new generation of state of the art tools (diagnostic tools, drugs, insecticides) and knowledge through novel research strategies in basic science and creative multidisciplinary approaches [17–19].

## The aim of Ethiopian government in controlling malaria transmission

The scaled-up use of long-lasting insecticidal nets (LLINs), indoor residual spraying (IRS) and treatment of cases with artemisinin-based combination therapy (ACT) contributed to the reduction of malaria transmission in the globe in general and in Ethiopia in particular in the past decade [20, 21]. Cognizant of the declining trend of the disease and the millennium health development goals, Ethiopia aims near zero malaria deaths, reduced malaria cases by 75% from baseline of 2013, and malaria eliminated in selected areas by the year 2020 and beyond [21]. To achieve these aims, there is a need for accurate epidemiological and entomological evidence that reveal the highest possible fraction of *Plasmodium* infections in the target human population and the dynamics of the vectors, respectively. Accurate diagnosis remains essential to target anti-malarial drugs, reduce transmission and enable management of other febrile illnesses. A declining level of transmission demands new diagnostic strategies and active case detection [22].

## Resources needed to control malaria transmission

### Improved diagnostic facilities

Improved diagnostic facilities provide timely, accurate and reliable results to support diagnosis, outbreak investigations, confirm clinical diagnoses, conduct accurate infectious disease surveillance, and direct public health care policy [23, 24]. Quality laboratory diagnosis depends on adequate and improved diagnostic facility, which requires adequate physical infrastructure and supplies of materials and reagents, uninterrupted electricity and running water, trained personnel, policy and strategic plan and synergy with clinical and research services [23–26]. Accurate diagnosis is essential not only to target anti-malarial drugs, but also to enable effective management of other febrile and infectious diseases.

Microscopy and rapid diagnostic tests (RDTs) are being used for the diagnosis of malaria in public health care facilities of Ethiopia. However, they fail to detect low level of parasitaemia and subpatent infections that contribute as reservoirs for subsequent transmissions [19, 27]. Most malaria vectors are members of morphologically indistinguishable groups of species or cryptic species which require advanced molecular methods such as the species specific polymerase chain reaction (PCR) [28]. Molecular diagnostic methods are also useful to determine the blood meal sources (human biting behaviours), *Plasmodium* sporozoite infection status and insecticide resistance status of the malaria transmitting *Anopheles* mosquitoes [29–32], which are important determinants of malaria transmission but are usually carried out in academic researches. In the era of intensified malaria control towards elimination of the disease, accurate diagnosis of cases and the vectors using the most reliable methods remains among the top priorities. To this end, the country requires establishment of quality assured and improved diagnostic facility in all its major malarious areas.

### Sound epidemiological and entomological evidence

In addition to political, financial and scientific considerations, a decision to control and consequently eliminate malaria must depend on a sound knowledge of the current prevalence of the disease by individual *Plasmodium* species, the distribution and behaviour of individual vector species, knowledge and socioeconomic status of risk groups and pattern of drug as well as insecticide resistance that meet local circumstances [33]. Sound epidemiological evidences can be obtained from health facility, school, sentinel site, cross-sectional and mobile clinics through standardized well thought surveys. The *Anopheles*–Human–*Plasmodium* interactions can be documented by employing either sentinel site or cross-sectional studies. Integration of research with customary work and strengthening of manpower doing both epidemiological and entomological work is required to reach the goals.

#### Health facility survey (HFS)

HFS captures basic epidemiological data on patients presented to health facilities such as hospitals, health centres, clinics and community health posts in a given time table. Evidences from such surveys include number of suspected patients, number of diagnosed cases, numbers of confirmed cases, number of inpatient malaria cases, number of inpatient malaria deaths, malaria test positively rate, percentage of *P. falciparum* or *P. vivax* or *P. falciparum* and *P. vivax* mixed infection and percentage of suspected malaria cases receiving a diagnostic test (Table 1).

**Table 1:** Strengths and benefits of the different types of malaria surveys

Type of malaria survey	Benefits and strengths
Health facility survey	Efficient means to collect basic epidemiological data such as numbers of malaria suspected patients, diagnosed (rapid diagnostic test, microscopy, molecular) cases, confirmed cases, deaths, impatient cases, impatient deaths and indictors such as test positivity rate, case incidence and mortality rate on a large number of cases [21, 35] Relatively cheap and easy method to collect longitudinal data on a large number of patients at different spatial and temporal scales [34] Provides temporal and spatial trends of the different malaria causing *Plasmodium* (*Plasmodium falciparum*, *P. vivax*, *P. ovale* and *P. malariae)* parasites Provide temporal (daily, weekly, monthly and yearly) and spatial trends of malaria cases, admissions and deaths by age and sex [21]
School-based survey	Capture temporal and spatial dynamic of malaria transmission which is inherently heterogeneous (unstable, seasonal, and linked to environmental variables such as altitude and rainfall) in Ethiopia [38] within a small and defined geographic scale Help to monitor and evaluate malaria control tools [38] Can help to trace focal outbreaks/epidemics
Sentinel site survey	Provides a comprehensive and longitudinal data on malaria and its vectors [39] Serves to evaluate efficacy of anti-malarial drugs and insecticides [39] Captures data related to malaria morbidity, mortality, diagnosis and treatment in a predetermined time [27, 39] Keeps the timeliness of reporting data [39] Helps to detect malaria outbreaks [27] Informs routine program decision making on commodity stocks and malaria burden in catchment areas [27]
Cross-sectional survey	Provides a comprehensive knowledge on coverage levels of preventive interventions (ITNs and IRS), fever case management practices, health-seeking behaviours, health status (under five mortality rate and anaemia), and parasite prevalence for clinical and subclinical malaria [34] Enables to identify most affected populations, trends of cases and deaths Helps to assess disease burden with parasite prevalence as the primary metrics Helps to respond to malaria epidemics and direct resources to populations most in need [33]
Mobile clinic survey	Inform and prevent the spread of endemic and emerging diseases, in addition to malaria, in seasonal workers’ home-, employment- and travel route-communities [41, 42] Prevent establishment of disease transmission in new and previously disease free areas [41, 42] Document disease burden associated with seasonal workers [41, 42] Inform routine program decision-making on commodity stocks [41, 42]
Vector survey	Generate data on the occurrence of species of malaria transmitting *Anopheles* mosquitoes [34] Inform human biting hours and preferred biting places of vectors for effective use of vector control interventions [34] Document spatial and temporal malaria transmission intensity; the entomological inoculation rate (EIR) [34] Document the insecticide susceptibility status of malaria vectors which is the most available control strategy Inform routine programme decision-making on the best vector intervention strategy

The Health Extension System in Ethiopia proves a unique opportunity for continuous surveillance throughout the country. Each *kebele* (lowest administrative unit) has a health post staffed by health extension workers that routinely test for malaria using RDTs. Although HFS provide longitudinal data on a large number of patients at different spatial and temporal points [21], they may underestimate asymptomatic and subpatent infections [34]. Such surveys represent only a fraction of all malaria cases occurring in a community, and are often biased in that the attendees may live closer to the facilities and have better access to medicines and a range of services and economic opportunities. Hence, HFS data may not represent the actual disease transmission in a given area [35]. In spite of its limitations, it is useful to show the temporal trend of the diseases if documented adequately.

#### School-based survey (SBS)

SBS is useful in areas where households are not easily accessible and resources are limited. SBS can help to estimate seasonality and prevalence (symptomatic, asymptomatic and subpatent infections) of malaria. In Ethiopia, primary schools are available in each community and hence can serve to describe community and area specific prevalence of the disease. In addition, SBS can help to determine haemoglobin level, nutritional status, and ownership and use of malaria intervention methods. Sub-Saharan Africa takes over 90% of the global malaria burden and children are the most affected [36, 37]. Therefore, schools can serve as centres to improve the lives and performance of children. In addition, it helps to evaluate performance of disease control programmes, to teach disease control tools, to undertake observed chemotherapy and evaluate drug/vaccine efficacy [38].

#### Sentinel site survey (SSS)

SSS provides a comprehensive and longitudinal data on malaria and its vectors and serves to evaluate efficacy of anti-malarial drugs and insecticides [39]. It captures data related to malaria morbidity, mortality, diagnosis and treatment in a predetermined time. It also keeps the timeliness of reporting data which can even be improved through integrating with short message services of mobile phones. SSS helps to detect malaria outbreaks through regular analysis of data and informs routine program decision making on commodity stocks and malaria burden in catchment areas [27].

#### Cross-sectional survey (CSS)

A CSS mainly involves administration of a questionnaire and collection of blood samples from household members in a target area. CSS involve suspicion, confirmation, investigation and reporting of transmission and provide knowledge on the epidemiology of the disease. It enables to identify most affected populations, trends of cases and deaths and also helps to assess impact of control measures. It also helps to direct resources to populations most in need and respond to epidemics [35]. The indicators to be generated include coverage and use of preventive interventions (LLIN and IRS), fever case management practices, health-seeking behaviours, health status (under five mortality rate and anaemia), and parasite prevalence for clinical and subclinical malaria cases. CSS can be undertaken at a local or national scale. In Ethiopia, the national cross-sectional surveys include Demographic Health Surveys and Malaria Indicator Surveys that provide a large amount of information, and are generally representative of the population. However, they are expensive and not performed frequently, have limited ability to capture data on malaria morbidity and do not monitor trends over shorter periods or on a fine spatial scale [34, 40].

#### Mobile clinic surveys (MCS)

In Ethiopia, establishment of mobile clinics remains a priority to treat seasonal workers who move to and from malaria endemic areas in search of employment. Work at mobile clinics can prevent the spread of several endemic and emerging diseases, including malaria in the home, employment and travel, and also prevent the establishment of disease transmission in new and previously disease free areas [41, 42]. Such clinics do help in the documentation of disease burden associated with seasonal workers. Mobile clinics should be considered an integral part of the envisaged malaria control and elimination strategy in the country.

#### Vector surveys

Active surveys of immature and adult *Anopheles* mosquito are useful to design sound vector control strategies. Larval survey is carried out in a variety of surface water collections ranging from lakes, swamps, marshes and rice fields to tree holes, hoof- and footprints to characterize vector breeding habitats and to monitor and evaluate the impact of vector control interventions. The important indicators from such surveys include occurrence, breeding seasons, density and preferred breeding habitats and insecticide susceptibility status of malaria vectors in a given area [43–45]. Preferably, vector surveys should be done simultaneously with epidemiological surveys or surveillance.

Adult *Anopheles* mosquitoes are surveyed to determine occurrence, seasonal density, distribution, resting and feeding behaviour, biting hour, human biting rate (HBR), entomological inoculation rates (EIR) per unit of time and insecticide susceptibility/resistance status of vectors and effectiveness of vector control tools. The entomological surveys can provide a rich source of data that dictate the time, place and type of malaria vector intervention for decision makers [10, 45–48]. To this end, employment of qualified entomologists to all malarious areas remains a top priority.

### Drug and insecticide resistance evaluation and monitoring system

Anti-malarial drug resistant *Plasmodium* parasites and insecticide resistant *Anopheles* vectors are critical challenges and are central to the planning and implementation of effective case treatment and insecticide (LLINs, IRS, Larviciding, Chemical Repellents, Insecticide treated Curtains) based vector control programmes. Furthermore, malaria control programmes are constrained by scarcity of trained personnel undertaking routine drug resistance and insecticide resistance evaluation/monitoring activities. As a result, drug resistant parasites and insecticide resistant vectors are mostly detected once operationally when significant increases in disease transmission occur [49]. Continuous drug efficacy surveillance makes the basis for ministries of health for possible replacement or prepare rational treatment strategies and policies [39]. Drug efficacy surveillances need to be undertaken regularly in representative sites. Possible new infection (different parasite strain) or recrudescence (same parasite strain) infections need to be genotyped as indicators of drug efficacy in selected routine health systems [39]. Globally, artemether-lumefantrine is efficacious against uncomplicated *P. falciparum*, but its efficacy might be lower in young and underweight children as a result of poor immunity and malnutrition [50]; this requires adequate studies. The growing evidences of *P. vivax* resistance to chloroquine in Ethiopia [51–53] also requires detailed studies before decision-making.

In order to minimize the increasing trend of insecticide resistance in malaria vectors, the WHO and its partners developed a global plan for insecticide resistance management (GPIRM). The basis of the plan is the building of capacity and systems for basic epidemiological and entomological monitoring, including bioassay for the insecticide susceptibility of vectors to insecticides in order to delay further development of resistance [54]. The insecticide-resistance status of local vectors must be determined before selecting the insecticide of choice [10]. Thus, there is a need to have a strong system that manage drug resistance and insecticide resistance [55].

The rapid decrease of malaria burden in several African countries, such as Equatorial Guinea, Burundi, Ivory Coast, Malawi, and Kenya, is in part attributed to the large scale use of LLINs in the presence of moderate-to-high pyrethroid resistance in the major vectors. The WHO classification of insecticide resistant mosquitoes refers to vector population mortality < 90% in 24 h following exposure to the insecticides in standardized bioassays [56] is used in surveillance. However, absence of mortality in such bioassays does not imply a complete absence of mortality. The phenotypic expression of resistance and the resistance level are dependent upon environmental variables like temperature, rainfall/moisture, vector behaviour, food, blood meal source and pre-existing pesticide exposure. Mosquitoes that are resistant at early age become more susceptible to insecticides when aging and reduce the epidemiology as most vectors of malaria are indeed old females. Hence, the insecticides may still be efficient at reducing the infectious vector population and thus malaria transmission. The indices of standardized WHO insecticide resistance assays and other related studies do not show epidemiological consequences of resistance and that entomological efficacy of vector controls may not directly correlate with epidemiological efficacy. Thus there is a need to investigate the impact of insecticide resistance on the epidemiology of malaria beyond immediate vector mortality [16, 57, 58].

Regular vector surveys are needed to be undertaken and be considered in the routine malaria surveys. Vector surveys also need to be integrated in the routine malaria surveillance systems such as malaria indicator surveys and demographic health surveys. *Anopheles arabiensis* is considered to be the primary malaria vector, with *Anopheles pharoensis*, *An. nili* and *An. funestus* serving as secondary vectors in some localities of Ethiopia. A recent study in a highland village of southern Ethiopia [59], revealed *P. falciparum* circumsporozoite protein positive *Anopheles demeilloni,* which had not previously been recognized as a malaria vector in the country. This incites the need for incrimination of the available species of *Anopheles* mosquitoes in view of the envisaged malaria control and elimination strategy in the country.

## Early-detection and early-warning system

Better information about the timing and locations of malaria epidemics would allow for more accurate targeting of resources for malaria prevention, control, treatment and elimination. Therefore, malaria surveillance is important for early detection of epidemics. In addition, there is a need to undertake continued environmental monitoring and seasonal climate forecasting as a major component of malaria early-warning system. In order to develop a working early-detection and early-warning systems, it is essential to first understand the underlying patterns and scale of malaria occurrence in both time and space [60]. To this effect, it is paramount important to undertake vulnerability monitoring, environmental monitoring and sentinels case surveillance or cross-sectional surveys [61]. As the transmission of malaria may not be synchronized over a wider geographical scale [60] early-detection and early-warning systems should be instituted at a narrow geographical scale which is to be determined on the basis of sound epidemiological and entomological studies. The early-detection and early-warning systems should also be integrated with dynamic online monitoring products such as climate conditions associated with malaria that are automatically updated when new data are available [62].

## Conclusion and the way forward

Ethiopia has embarked a strategy to bring a near zero malaria deaths, reduced malaria cases by 75% from baseline of 2013, and malaria eliminated in selected areas by the year 2020 [21]. To sustain its current achievements of reduced malaria cases and deaths and also to attain its new strategy, the country requires quality assured diagnostic laboratories in all health care facilities of malarious area. In addition, the existing microscopic and rapid diagnostic test based diagnosis methods of the disease need to be upgraded to modern molecular techniques which are both sensitive and specific. The modern molecular techniques enable to undertake improved diagnosis of *Plasmodium* parasites (both in humans and *Anopheles* vectors) and malaria transmitting *Anopheles* species. Improved diagnostic facilities provide reliable data for sound epidemiological and entomological evidences that dictate the achievements of ongoing control and elimination activities in the target population. If Ethiopia is going the control malaria deaths to near zero and ultimately eliminate the disease, laboratory and epidemiological services need to be expanded to all malarious areas and decentralized.

## Abbreviations

LLIN: long-lasting insecticide treated net
IRS: indoor residual spraying
ACT: artemisinin-based combination therapy
RDT: rapid diagnostic test
PCR: polymerase chain reaction
HFS: health facility survey
SBS: school based survey
SSS: sentinel site survey
CSS: cross-sectional survey
MCS: mobile clinic survey
HBR: human biting rate
EIR: entomological inoculation rate
WHO: world health organization
GPIRM: global plan for insecticide resistance management
